# Vascularization potential of a dermal skin substitute material (Biodegradable Temporizing Matrix) by proangiogenic growth factors and ASC - an in ovo study

**DOI:** 10.1007/s10856-025-06982-4

**Published:** 2025-12-13

**Authors:** Maximilian Diemer, Sarah Strauß, Vesna Bucan, Maike Anna Busch, Nicole Dünker, Peter M. Vogt, Nicco Krezdorn, Frederik Schlottmann

**Affiliations:** 1 Department of Plastic, Aesthetic, Hand and Reconstructive Surgery, Hannover Medical School, Hannover, Germany; 2 Institute of Anatomy II, Department of Neuroanatomy, Medical Faculty, University of Duisburg-Essen, Essen, Germany; 3 Department of Plastic and Breast Surgery, Roskilde University Hospital, Roskilde, Denmark

## Abstract

In the treatment of severe burn injuries, autologous skin transplantation is increasingly being supplemented by synthetic dermis substitute materials. Novosorb® Biodegradable Temporizing Matrix (BTM) is a polyurethane foam used in a surgical procedure that currently requires a period of up to 21 days for successful neovascularization and integration, which is associated with a longer inpatient treatment. The objective of this study was to assess the efficacy of the growth factors EPO, FGF, PDGF, VEGF and adipogenic stem cells (ASC) in shortening the time required for BTM grafting and vascularization. BTM containing growth factor and/or ASC was grafted onto to the chorioallantoic membrane (CAM) in different configurations. The average vascular growth of the BTM in 9 different experimental groups was analyzed in comparison to the control group. After 7 days, the experiment was terminated, and the vascularization of the BTM was evaluated by macroscopic image analysis with ImageJ/Fiji, along with histological HE staining and immunohistochemical staining for vascular-specific factors. Successful grafting and vascularization of the BTM in ovo were achieved for the first time. The addition of growth factors and ASC increased the average vascularization of the BTM and the entire CAM. All experimental groups showed promising vascularization patterns, with the BTM + ASC and BTM + PDGF + ASC groups excelling. Differentiation of ASC was not induced in combination with BTM or growth factors. BTM vascularization is improved by proangiogenic growth factors and ASC, which can form the basis for clinical strategies aimed at shortening the inpatient treatment of severely burned patients.

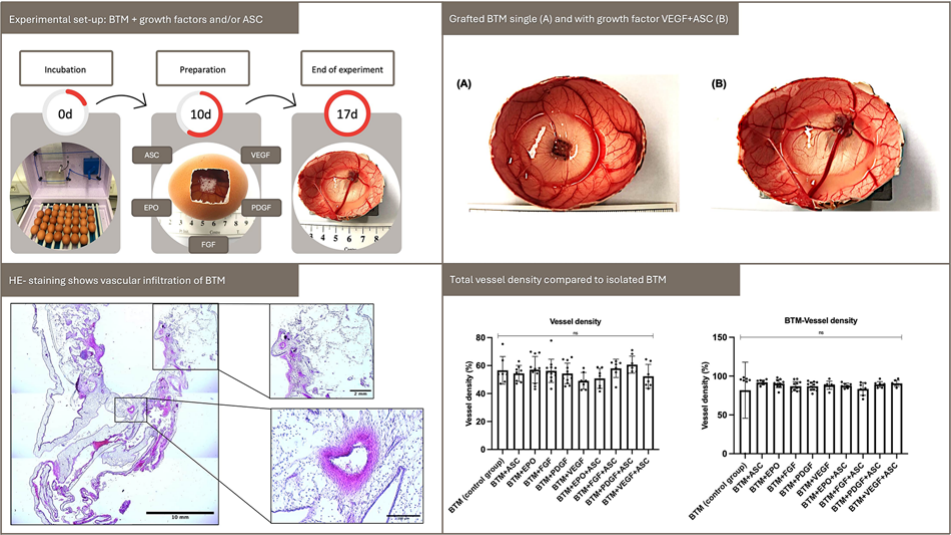

## Introduction

The care of severely burned patients represents a major interdisciplinary challenge in clinical practice [1]. According to a 2018 World Health Organization report, 180,000 people die every year as a result of burns [2]. Severely burned patients require long-term intensive care treatment in specialized burn centers, focusing on burn specific complications such as multi-organ failure, sepsis, burn shock and respiratory disorders [3, 4]. Plastic-reconstructive surgery aims to restore the integrity of the skin as soon as possible, as prolonged wound healing is associated with increased mortality [5, 6]. Autologous skin grafting remains the gold standard in skin reconstruction but shares the main disadvantage of donor site morbidity [7, 8]. Especially in severely burned patients with an extensive total body surface area (TBSA) affected, donor sides are often limited [9]. Autologous skin grafting still cannot reach the aesthetic and functional quality of original skin, particularly when the defect extends beyond the dermis [10]. To address these shortcomings, efforts over recent decades have focused on developing allogeneic, xenogeneic, synthetic or mixed materials to create ideal skin substitutes for treating severely burned patients [11]. Skin substitutes can be classified according to permanence, layering, cellularity, and the recipient site [12]. Ideally, a skin substitute should consist of both epidermal and dermal components, resist infection and rejection, integrate and vascularize rapidly, be cost effective and readily available, and provide durable wound closure that withstands mechanical shear forces while maintaining maximum function [13, 14].

Synthetic skin substitutes appear superior due to their low immunogenicity, optimized mechanical properties, cost efficiency in production, and their individualization in clinical applications [15, 16]. There are currently a plethora of synthetic skin substitutes available. The most commonly used dermal skin substitutes in clinical practice, the biosynthetic Integra® (Life Sciences, Plainsboro, NJ, USA) and the fully synthetic NovoSorb® Biodegradable Temporising Matrix (BTM) (PolyNovo Biomaterials, Port Melbourne, VIC, Australia) were compared in an experimental trial with athymic nude mice [17]. BTM demonstrated superior neovascularization kinetics and skin cell infiltration, potentially due to its higher resistance to infections [18]. BTM is indicated for second- or third-degree burns, as well as complex or traumatic wounds [19]. BTM was approved in Europe in 2019 and consists of a two-layered polyurethane foam with an outer sealing membrane [20]. In clinical routine, BTM is applied in a two-stage surgical procedure. After debridement, BTM is placed onto a macroscopically clean wound bed and the wounds can be temporarily covered using vacuum therapy [21]. Once vascularization becomes visible, indicating integration, definitive defect coverage is performed using autologous split-thickness skin grafting [22]. One of the main disadvantages of BTM is the time required for integration, as it takes ~21 days to achieve neovascularization and integration, according to the manufacturer [19]. Therefore, shortening this period would significantly improve patient outcomes and lead to more optimized and cost-effective treatment [23].

Adipose-derived stromal cells (ASC) derived from autologous adipose tissue, along with certain growth factors play a key role in regenerative medicine due to their proven long-term wound healing effects [24–26]. The promising interaction between ASC and growth factors was demonstrated by Zhou et al. in an animal model, where enhanced expression of vascular endothelial growth factor (VEGF) was observed following ASC injection into burn wounds [27].

The aim of this proof-of-concept study was to investigate the vascularization and angiogenesis of BTM in ovo using the in ovo chorioallantoic membrane (CAM) model. To enhance the neovascularization and integration of BTM, ASC and recombinant growth factors such as erythropoietin (EPO), fibroblast growth factor (FGF), platelet-derived growth factor (PDGF) and VEGF were applied in ovo. The long-term clinical goal remains to accelerate vascularization of BTM in vivo, thereby shortening inpatient treatment and reducing the associated costs.

## Materials and methods

### Cell culture

ASC used in the present study were isolated from skin tissue donated by a 38-year-old Caucasian female after abdominoplasty. Cell harvesting was conducted in accordance with the Declaration of Helsinki, and the protocol was approved by the Ethics Committee of Hanover Medical School (approval number: 3475-2017 and date of approval: 15 February 2017). All patients involved in this study provided written consent. ASC were isolated and characterized following standardized protocols described elsewhere [28]. ASC were cultured in phenol red-free Dulbecco’s Modified Eagle Medium (DMEM/F-12) (Thermo Fisher Scientific, Waltham, USA), supplemented with 10% (v/v) fetal bovine serum (Cell Line Services, Eppelheim, Germany), 0.1% (v/v) ascorbic acid-2-phosphate (Sigma-Aldrich, Merck, Darmstadt, Germany), and 1% (v/v) 10 mg/mL penicillin and streptomycin (Sigma-Aldrich, Merck, Darmstadt, Germany). Cells were incubated at 37 °C with 5% CO_2_ in a humidified atmosphere. Upon reaching confluence, cells were detached with 0.25% (v/v) trypsin/ethylene diamine tetra acetic acid (Gibco, Thermo Fisher Scientific, Waltham, MA, USA) and subcultured. ASC of passage 1 were used for all the following experiments. In a titration series, an optimal cell number of 25,000 ASC per egg was determined in advance based on the authors’ experiences using ASC cultured biomatrices, such as spider silk and collagen type I hydrogels. The ASC were resuspended in culture medium and applied to the matrices. A drop of cell suspension was then applied to BTM and incubated for 60 min at 37 °C with 5% CO_2_ in a humidified atmosphere.

### Recombinant growth factors

Recombinant growth factors were commercially available: human EPO (PeproTech, Thermo Fisher Scientific, Cranbury, NJ, USA), human FGF (Miltenyi Biotec, Bergisch Gladbach, Germany), human PDGF (Miltenyi Biotec, Bergisch Gladbach, Germany) and human VEGF (PeproTech, Thermo Fisher Scientific, Cranbury, NJ, USA). The final concentrations were selected based on published literature and the manufacturer’s instructions: 1 ng/mL EPO, 2 ng/mL FGF, 2 ng/mL PDGF, and 5 ng/mL VEGF.

### NovoSorb® Biodegradable Temporising Matrix (BTM)

NovoSorb® Biodegradable Temporising Matrix (BTM) (PolyNovo Biomaterials, Port Melbourne, VIC, Australia) is a three-dimensional, two-layer fully synthetic dermal skin substitute made of polyurethane. BTM used in this study was commercially available. According to the manufacturer’s specifications, the dermal component consists of a 2-mm thick polyurethane foam that biodegrades through hydrolysis. The outer surface has a non-biodegradable polyurethane sealing membrane that mimics a pseudo-epidermis to limit fluid loss. For the following experiments, the BTM was cut into 0.5 cm^2^ pieces under sterile conditions. The BTM pieces were not re-sterilized. Three days prior to the experiment, the BTM was soaked in cell culture medium to prevent drying of the CAM. Immediately before application to the CAM, the medium was aspirated from the BTM.

### CAM assays

In accordance with the German Animal and Welfare Law, CAM assays do not require ethical and legal approval by the local or government authorities. Nevertheless, the trial was carried out in close coordination with the animal welfare officer of Hannover Medical School. Table 1 provides an overview of the experimental groups used for the in ovo experiments.

**Table 1:** Overview of the experimental groups for the in ovo experiments

Experimental group number	Experimental approaches in ovo
1	BTM (control group)
2	BTM + ASC (25,000 cells per egg)
3	BTM + EPO (1 ng/mL)
4	BTM + FGF (2 ng/mL)
5	BTM + PDGF (2 ng/mL)
6	BTM + VEGF (5 ng/mL)
7	BTM + EPO (1 ng/mL) + ASC (25,000 cells per egg)
8	BTM + FGF (2 ng/mL) + ASC (25,000 cells per egg)
9	BTM + PDGF (2 ng/mL) + ASC (25,000 cells per egg)
10	BTM + VEGF (5 ng/mL) + ASC (25,000 cells per egg)

For each experimental group, ≥7 fertilized leghorn eggs (Geflügelzucht Horstmann, Stolzenau, Germany) were incubated for 10 days at 38 °C and 50% humidity in a fully automated surface incubator type 3000 (Siepmann, Herdecke, Germany). At embryonic development day (EDD) 10, eggs were transilluminated with a cold light source type KL200 (Leica Microsystems, Wetzlar, Germany) and a vascular branch was marked when fertilization occurred. Non-fertilized eggs were sorted out. After preparation a 1 mm hole into the air sac and a 0.5 cm^2^ shell window by a multifunctional tool (DREMEL Europe, Breda, Netherlands), a 0.5 cm^2^ piece of BTM was placed on the vascular branch of the CAM. Subsequent addition of growth factors and/or ASC was performed according to the experimental groups as shown in Table 1. Growth factors and ASC were titrated to make them available as a 5 µl solution and dripped onto the BTM. After sealing the basal hole and preparation window with Leukosilk® S (BSN medical, Hamburg, Germany), the eggs were incubated further without rotation. After 7 days of in ovo cultivation (EDD 17), samples were processed for analysis. For this purpose, after a minimum of 30 minutes of cooling with ice, starting from the basal hole, the egg was opened equatorially. The vascularization of the BTM was documented macroscopically and photographically, followed by image analysis as described below. The lower half of the egg, including the BTM and blood vessels, was dissected. Embryos were sacrificed by decapitation. At least 7 eggs per experimental group were analyzed, while 7 eggs were evaluated as a CAM control group on EDD 10.

### Histology

Samples were fixed in 10% (v/v) buffered formalin (Carl Roth, Karlsruhe, Germany) for 48 h, then dehydrated in a graded series of increasing alcohol concentrations, cleared in xylene (Carl Roth, Karlsruhe, Germany) and embedded in paraffin (Carl Roth, Karlsruhe, Germany) using a Epredia™ STP 120 Spin Tissue Processor (MICROM International, Walldorf, Germany). Sections of 14 µm were cut with a microtome (MICROM International, Walldorf, Germany), deparaffinized, rehydrated in a graded series of decreasing alcohol concentrations, and stained with hematoxylin/eosin (HE). For HE staining, samples were stained with 1% (v/v) hematoxylin according to Meyer (Merck, Darmstadt, Germany) for 5 min, rinsed with tab water for 10 min, and stained with 2% (v/v) eosin (Merck, Darmstadt, Germany) for an additional 2 min. To exclude osteogenic mineralization and adipogenic differentiation of ASC, slides were stained with oil red o as specific adipogenic dye and alizerin red as specific osteogenic dye according to standardized protocols. To exclude differentiation of the ASC in cartilage tissue, connective tissue or muscle tissue, alcian blue, herovici and trichrome stains were used. Bright-field microscopy using a Keyence BZ-8000K microscope (Keyence, Neu-Isenburg, Germany) and associated software was performed to view stained sections. As adverse differentiation was not observed, oil red o, alcian blue, herovici, trichrome, and alizerin red stains are not shown in the manuscript.

### Immunoflourescence

To examine the vascularization of BTM, samples from each experimental group were stained using indirect immunofluorescence. After dehydration and paraffin embedding following the above given protocols, samples were washed thrice in phosphate buffered saline (PBS) (Life Technologies, Darmstadt, Germany), permeabilized with 0.2% (v/v) Tritron X-100 (Carl Roth, Karlsruhe, Germany) for 4 min, and blocked with 2% (v/v) bovine serum albumin (BSA) (Sigma-Aldrich, Merck, Darmstadt, Germany)/PBS solution for 60 min. After washing again with PBS, primary antibodies were applied. As primary antibodies monoclonal mouse anti-Factor VIII antibody (abcam, Cambridge, UK; #ab78852), polyclonal rabbit anti-alpha smooth muscle actin (SMA) antibody (Merck, Darmstadt, Germany; #ABT1487) and monoclonal rabbit anti-prospero homebox protein 1 (PROX1) antibody (abcam, Cambridge, UK; #ab199359) were used. Primary antibodies were diluted in a ratio of 1:100 in 1% (v/v) BSA and incubated at 4 °C over night, followed by washing with PBS. Alexa Fluor^™^ 488 conjugated goat anti-mouse antibody (life technologies, Thermo Fisher Scientific, Waltham, MA, USA) and TexasRed^®^ conjugated goat anti-rabbit antibody (life technologies, Thermo Fisher Scientific, Waltham, MA, USA) were used as secondary antibodies. Secondary antibodies were incubated for 30 min. Sections were covered and counterstained with SlowFade^®^ Diamond Antifade Moutant with 4′,6-diamidino-2-phenylindole (DAPI) (life technologies, Thermo Fisher Scientific, Waltham, MA, USA) according to the manufacturer’s instructions. Inverse fluorescence microscopy was performed to view the stained sections using an IX83 microscope (Olympus, Tokyo, Japan) and corresponding software.

### Image analysis

Images of the opened egg with the centrally ingrown BTM were taken on EDD 17 using an iPhone camera (iPhone 8 Plus with 1920 ×1080 pixels; Apple Inc., Cupertino, CA, USA). Images were uploaded to the open-source Java-based ImageJ/Fiji 2.90/1.53t software (National Institutes of Health, USA). After pre-processing, a binary image was created. Within the region of interest, the vessel density was automatically calculated from the skeletonized vessel area/total area *100% by Vessel Density plugin (Supplementary Fig. 1). An analysis of the vascularization of the entire CAM including BTM and the isolated vascularization of the BTM were performed for each egg.

### Statistical analysis

As this is a proof-of-concept study, each experiment was carried out as a single copy. Data are expressed as mean ± standard deviation. GraphPad (GraphPad Prism version 10 (10.3.0)) was used to perform an one-way ANOVA analysis for statistical variance between the experimental groups. A *p*-value less than 0.05 was considered statistically significant.

## Results

**Fig. 1:**
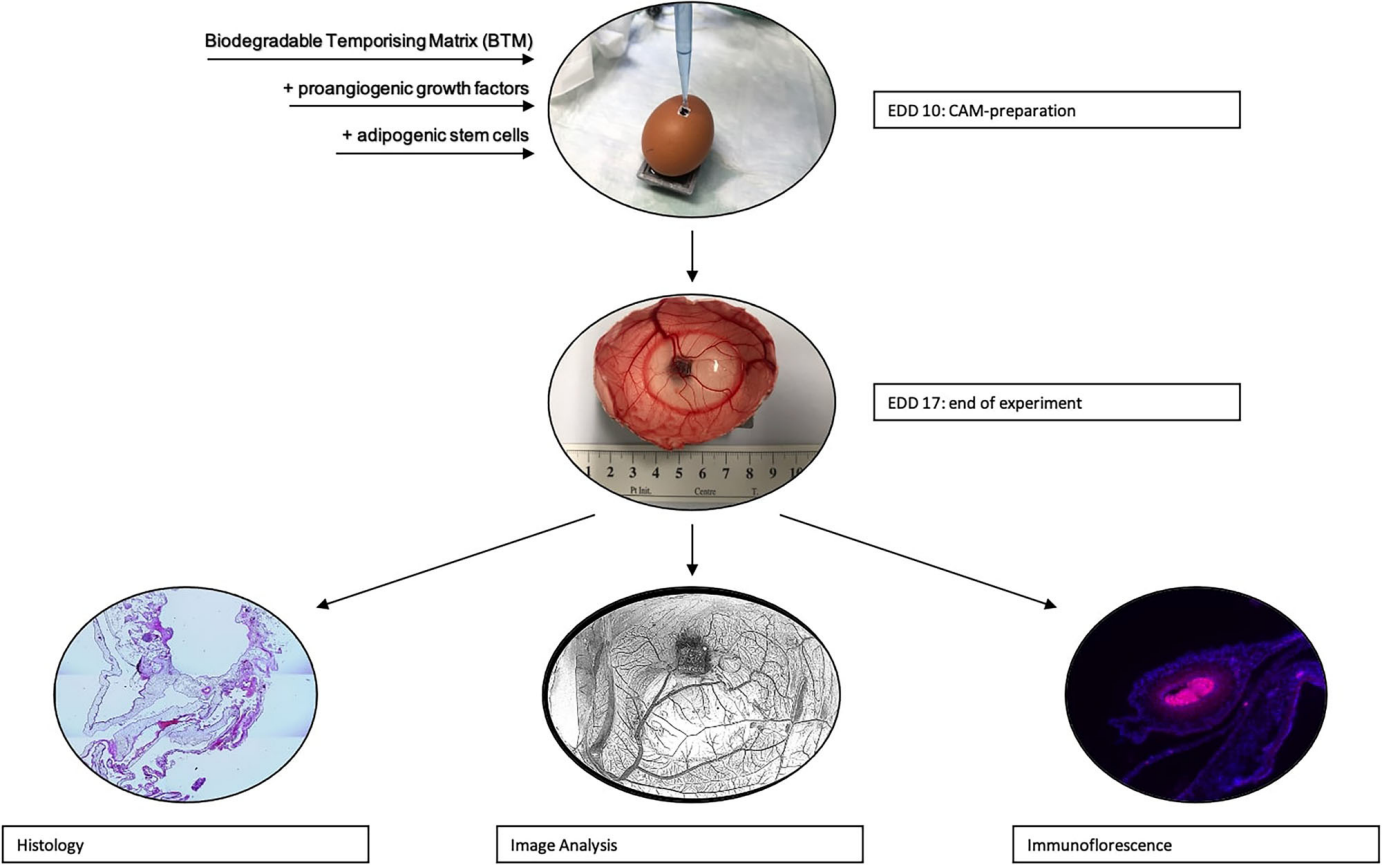
Experimental set-up. Embryonic developmental day (EDD) 10: The CAM was prepared via the smallest possible opening after the CAM was dropped, thereby creating an artificial apical air chamber. BTM was grafted, and growth factors and/or ASC were added depending on the experimental group. The openings were then closed, and eggs were re-incubated for an additional 7 days. EDD 17: The egg was opened horizontally, and an in situ macroscopic image of BTM within the CAM (including the resulting vessels) was immediately taken. The CAM was then excised and fixed in formalin for histological and immunohistochemical staining

The present proof-of-concept study explored the vascularization potential of BTM, a synthetic dermal substitute, when supplemented with selected proangiogenic growth factors as well as ASC. Figure 1 gives an overview of the experimental set-up using the CAM assay. A macroscopic and photographic evaluation of the vascularization was performed on embryonic developmental day (EDD) 17. The results were correlated with histologic and immunohistologic evaluations of vascular infiltration. A distinction was made as to whether the entire egg including the matrix was infiltrated by vessels and to what extent the matrix was infiltrated by vessels when viewed individually (Supplementary Fig. 1).

### Macroscopic observations: vessels penetrated the BTM matrix and were multiplied by the addition of growth factors and/or ASC

**Fig. 2:**
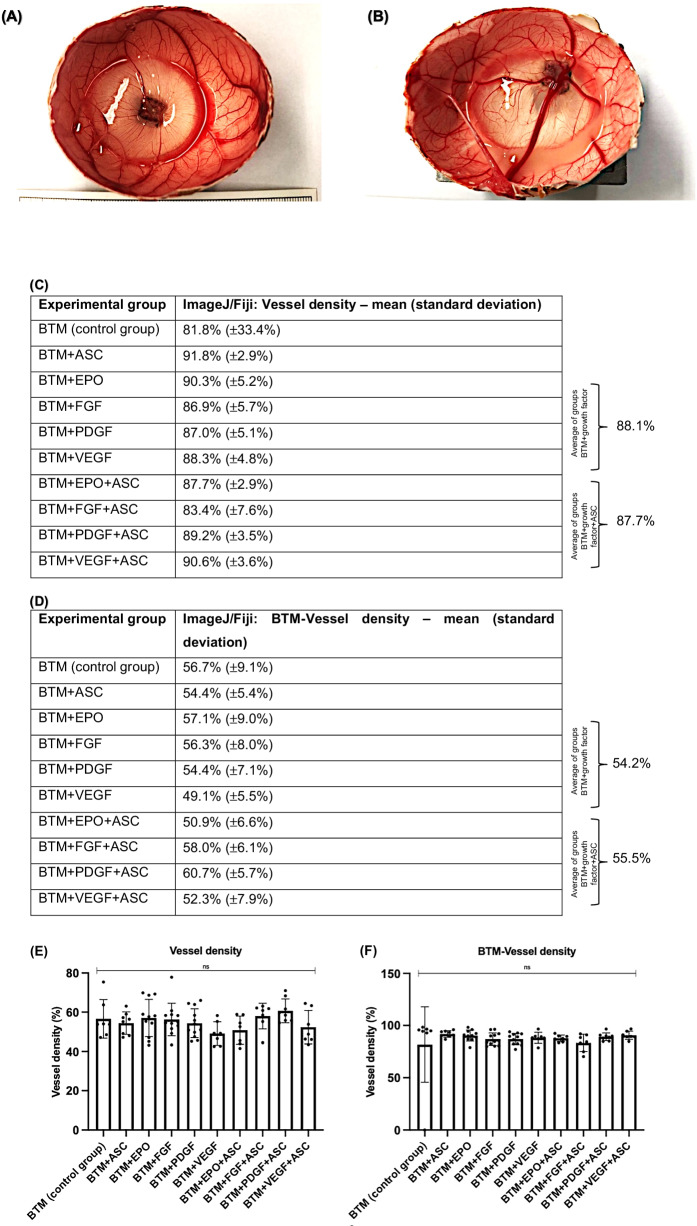
BTM grafting on EDD17 with CAM vessels vascularized differently based on macroscopic analyses. **A** Experimental group 1 (control with BTM only): The BTM is in the center of the CAM with sprouting vessels and a reddish discoloration of the matrix as a sign of vascularization after 7 days of in ovo incubation. **B** Experimental group 10 (BTM + VEGF + ASC): Noticeable difference in the number and diameter of the vessels as the BTM is infiltrated by at least 6 vessels. **C** Means and standard deviations of total CAM vascularization in the control group compared to the experimental groups based on macroscopic analyses of the images using ImageJ/Fiji. Seven eggs were analyzed per experimental group. Average values for all experimental groups treated with growth factors or growth factors and ASC are shown. **D** Means and standard deviations of CAM vascularization within BTM from the control group compared to the experimental groups based on macroscopic analyses of the images using ImageJ/Fiji. The experimental groups were the same eggs as in (**C**). Mean values are shown for all experimental groups treated with growth factors or growth factors and ASC. **E** Individual values of test groups and the respective vascularization (%) achieved from total vascularization analysis of the eggs, representing the mean values of (**C**). No significant differences between the groups were observed (*p* = 0.152). ns not significant. **F** Individual values of test groups and the respective vascularization (%) achieved total vascularization analysis of the eggs, representing the mean values of (**D**). No significant differences between the groups were observed (*p* = 0.800). ns not significant

According to the previously described experimental setup, growth factors were applied in the minimum effective concentration, either alone or in combination with ASC as shown in Table 1. The macroscopic evaluation of the equatorially opened eggs was carried out on EDD 17. Figure 2 shows examples of grafting and vascularization of BTM onto the CAM, demonstrating that the vessel density, as determined macroscopically, increased in the experimental groups (Fig. 2B) compared to the control group (Fig. 2A), depending on the administration of growth factors and/or ASC. The results are shown in Fig. 2C (complete vascularization analysis of the CAM) and Fig. 2D (isolated analysis of BTM vascularization within the CAM).

Starting with the analysis of overall CAM vascularization (Fig. 2C), the control group (experimental group 1) showed an average vessel density of 81.8% (±33.4%). For the experimental groups where growth factors (experimental groups 3–6) or growth factors and ASC (experimental groups 7–10) were applied, joint average value of 88.1% and 87.7% were calculated. Experimental group 2 (BTM + ASC) had the highest vascularization with 91.8% (±2.9%), followed by experimental group 10 (BTM + VEGF + ASC) (90.6% (±3.6%)) and experimental group 3 (BTM + EPO) (90.3% (±5.2%)). No conclusion can be drawn at this time about whether growth factors, ASC, or their combination is best for vascularization. Notably, FGF (experimental group 4) and FGF + ASC (experimental group 8) showed the lowest kinetics of vascularization (86.9% (±5.7%) and 83.4 (±7.6%)). However, FGF showed superior vascularization with the BTM. The standard deviation for the experimental groups ranged between 2.9% and 7.6%, with the exception of the control group, which had a standard deviation of 33.4%. Statistical ANOVA analysis revealed no statistically significant difference between the groups (*p* = 0.152) as shown in Fig. 2E.

Continuing with the isolated examination of BTM vascularization, the control group showed an average vessel density of 56.7% (±9.1%). The joint average value for all experimental groups where growth factors were added was 54.2% (experimental groups 3–6), and 55.5% for growth factors and ASC (experimental groups 7–10). The experimental groups 9 (BTM + PDGF + ASC (60.7% (±5.7%)), 8 (BTM + FGF + ASC (58.0% (±6.1%)) and 3 (BTM + EPO (57.1% (±9.0%)) exceeded the control group. The experimental group 6 (BTM + VEGF) had the lowest vascularized with an average value of 49.1% (±5.5%). The standard deviations in the individual test groups ranged between 5.4 and 9.1%. No statistically significant differences for BTM vascularization were detected between the groups (*p* = 0.800) as shown in Fig. 2F. A power analysis for both levels of vascularization indicated that, at a significance level of α = 0.05, a power of 0.80 and a medium effect size (f = 0.25), ~259 samples per group would be required to detect a significant difference.

When considering all test groups after a 7-day period, successful adhesion and vascularization of the BTM onto the CAM vasculature were observed (Supplementary Fig. 2). Further details on the correlation between the administration of growth factors and/or ASC and the different experimental groups are summarized in Fig. 2C, D.

### Histological evaluations confirmed the macroscopically observed trend of vascular enhancement of BTM by adding growth factors and/or ASC

**Fig. 3:**
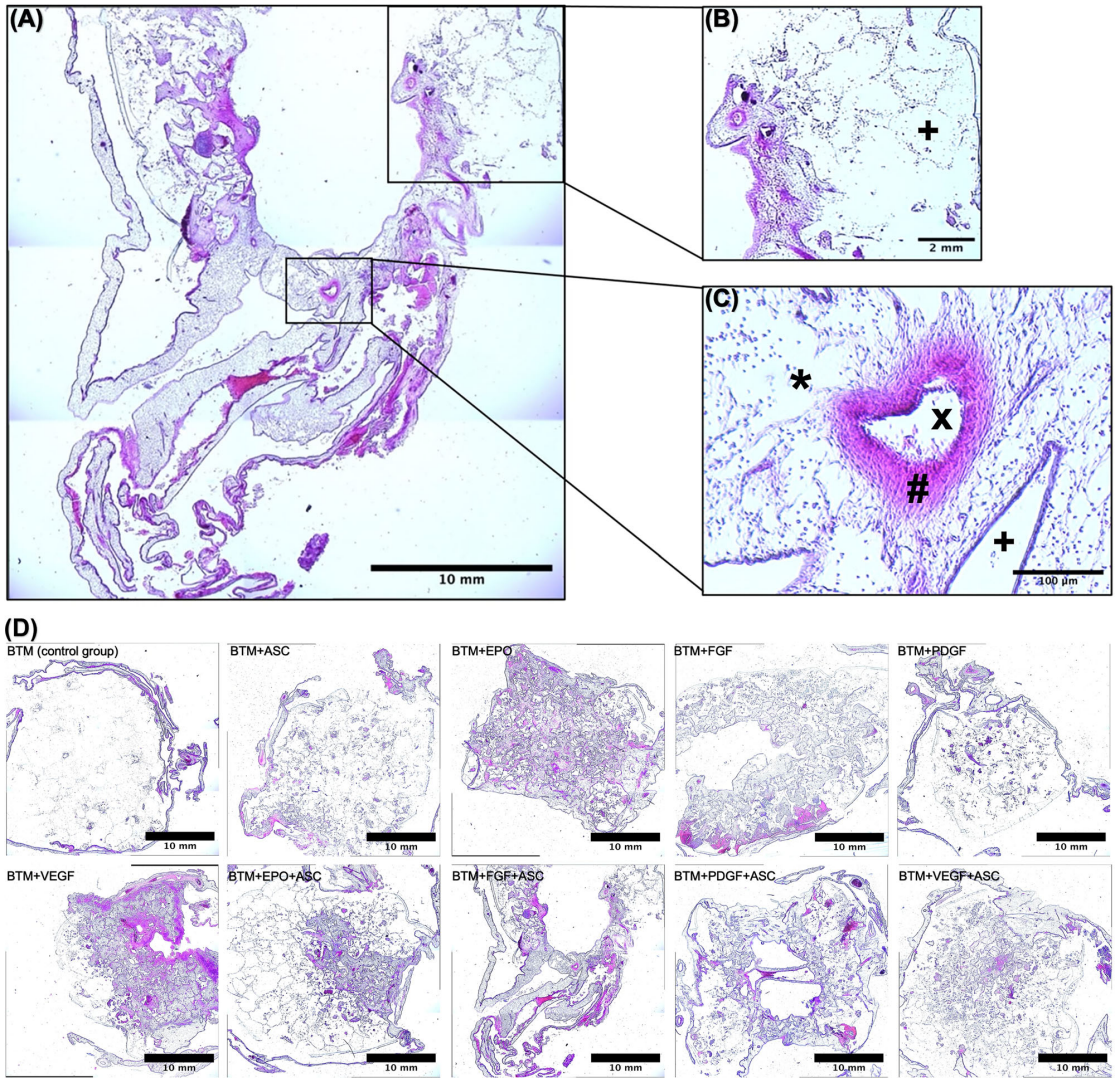
Cross-section HE staining showing varying degrees of vascular infiltration into BTM in the CAM model. **A** CAM section of the experimental group 8 (BTM + FGF + ASC), with bright grid-like BTM structures. In most areas, the BTM structure is covered by reddish and purple hues, typically indicating coagulated blood or leukocytes. A vascular lumen is cut in the center of the image, and other blood vessels of different calibers and thicknesses are visible. **B** 4× magnification of (**A**), enlarging the grid-like structure of BTM. Cells have accumulated on and within the BTM. Furthermore, a vessel surrounded by blood products is also seen. **C** 40× magnification to (**A**), showing a vessel with typical wall stratification and lumen containing cell nuclei. +BTM, *(endothelial) cells, ^x^vascular lumen with endothelial cells, ^#^vessel wall. **D** A representative HE-stained section from each experimental group, showing vessels differently distributed within the matrix depending on the experimental group

To further support the macroscopic observations, histological analysis of the HE sections was performed. Figure 3 provides an example from experimental group 8 (BTM + FGF + ASC) showing an overview (Fig. 3A), separate BTM structure (Fig. 3B) and BTM-infiltrating vessels (Fig. 3C). The BTM serves as a scaffold for tissue grafting without forming a fibrous capsule or showing sign of a foreign-body reaction, which is undesirable. As seen in Fig. 3D, vessels occupy both the BTM and the interstitial spaces between the BTM pillars. Each group exhibited varying degrees of vascularization and tissue infiltration into the BTM.

### The influence of ASC on vascularization

When measuring total vessel density with ImageJ/Fiji, a difference was observed between the joint average values of the BTM + growth factors group (88.1%) and the BTM + growth factors + ASC group (87.7%). The mean vascular density of the BTM + ASC group was 91.8% (±2.9%), indicating that this experimental group showed the highest vascularization when analyzed using this method. Within the BTM, vascularization averaged 54.2% in the BTM+growth factors group, increasing to 55.5% in the BTM + growth factors + ASC group. The BTM + ASC group, with an average vascularization value of 54.4% (±5.4%), fell between these two values. It can be concluded from this that ASC positively influence vascularization. HE staining and immunohistochemical staining support this conclusion, as pronounced patterns of vascularization were observed in the groups with ASC.

### Differentiation of ASC

Since ASC can differentiate into bone, cartilage, muscle and adipose tissue, histological examination was performed for fat tissue (oilred O staining), bone tissue (alicerin red staining), cartilage tissue (alcian blue staining), muscle tissue (trichrome staining), and connective tissue (herovici staining). Overall, no differentiation of the ASC was observed in any experimental groups or control groups after 17 days. For clarity, the data are not shown in this manuscript. Therefore, differentiation of the ASC by BTM or the addition of EPO, FGF, PDGF and VEGF can be excluded at this stage.

### Immunohistochemical survey

**Fig. 4:**
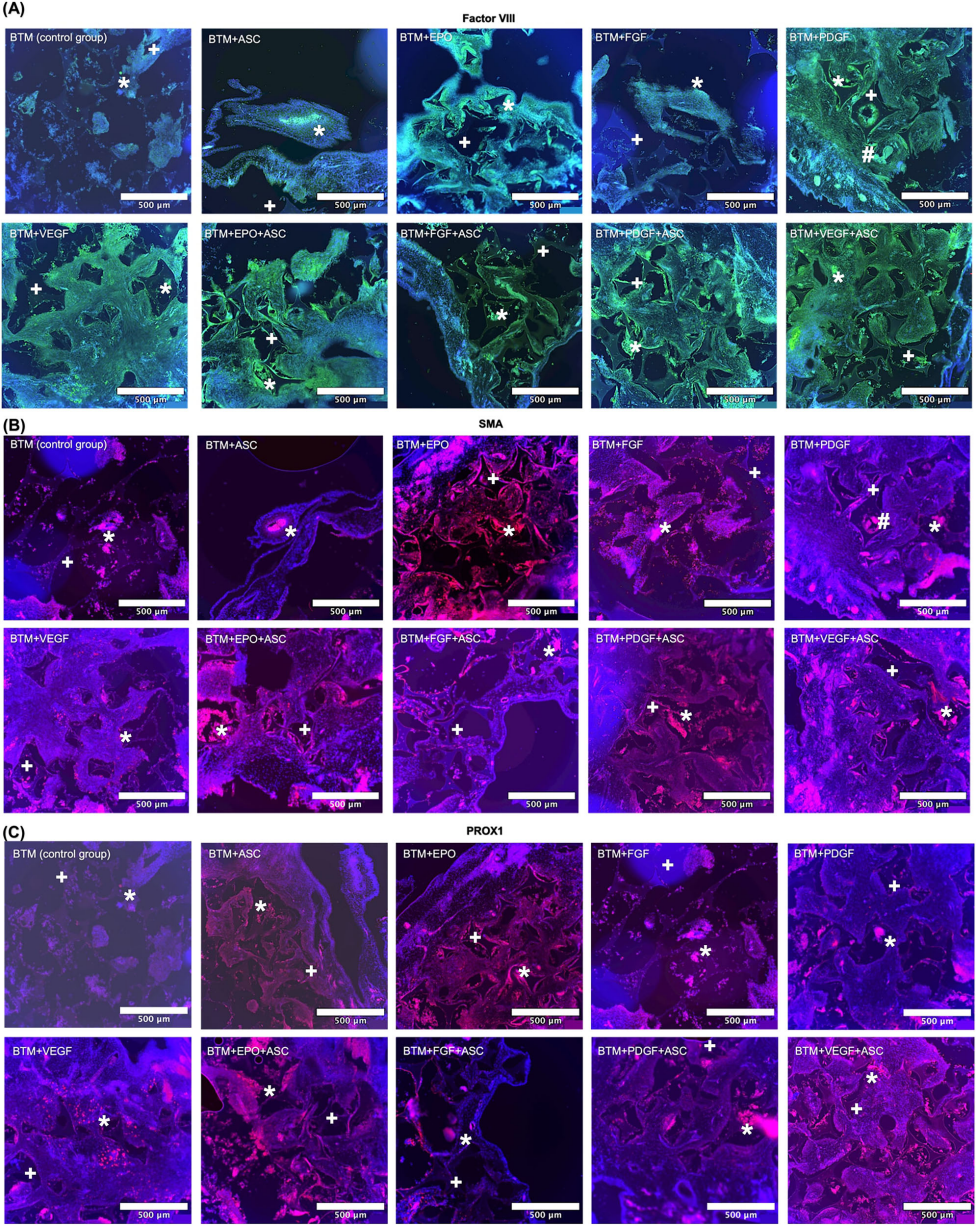
Illustration of all experimental groups stained with antibodies against Factor VIII, SMA and PROX1. Nuclei are counterstained with DAPI in blue fluorescent. **A** In the Factor VIII staining, endothelial cells appear green fluorescent. To varying degrees, endothelial components can be seen infiltrating the BTM in all experimental groups. In the cross-section of experimental group 5 (BTM + PDGF), a vascular lumen and endothelial cells within the BTM can be clearly seen. **B** In the SMA staining, endothelial cells appear red fluorescent. All sections shown here are vascularized to varying degrees, as indicated by the presence of endothelial cells. Experimental group 5 (BTM + PDGF) best demonstrates the infiltration of the BTM by a vessel. No BTM structure is identifiable in the sectional view of experimental group 2 (BTM + ASC), suggesting that this was completely infiltrated by endothelial cells. **C** PROX1 staining identifies lymphatic endothelial cells as red fluorescent. Lymphatic activity was found in all experimental groups, with varying degrees of expression. No pattern could be recognized in the cross-sections to indicate whether certain growth factors or ASC induce a lymphatic response to a greater extent. *(endothelial/lymphatic) cells, ^+^BTM, ^#^vessel wall

Immunohistochemical staining was used to further investigate the results from ImageJ/Fiji (Fig. 2) and HE staining (Fig. 3) regarding vessel growth. Primary antibodies against Factor VIII, SMA and PROX1 were used to visualize endothelial cells as well as lymphatic endothelial cells, and the results are shown in Fig. 4. For clarity, the groups are presented consecutively from left to right per immunohistochemical staining, analogous to Table 1. Due to the staining properties of the biomaterial, the BTM only provides an observational and not a quantitative possibility of evaluation using ImageJ/Fiji.

### Factor VIII

Factor VIII is a specific endothelial cell marker that allows conclusions to be drawn about angiogenesis and serves as the first suitable method for confirming earlier results. For example, in experimental group 5 (BTM + PDGF), the rhomboid structure of the BTM with infiltrating endothelial cells and a vascular lumen, can be clearly seen. Green-fluorescent cells were visible in all experimental groups, confirming their identity as endothelial cells. The images of all experimental groups show that endothelial cells adhere to the BTM from both the inside and the edges, as already observed in the HE staining. The presence of endothelial cells was observed in all experimental groups within the selected image area, albeit to varying degrees.

### SMA

Alpha smooth muscle actin (SMA) is a protein found in smooth muscle cells, making it suitable for visualizing endothelial cells in blood vessels. This supports the results of the HE staining and the Factor VIII antibody staining. As with the previous antibody staining, endothelial cells were visualized in all experimental groups, adhering to and within the BTM. The experimental group 5 (BTM + PDGF) again exemplifies how a preserved vessel has become established within the BTM. Similar to Factor VIII staining, the vascularization of this group appeared most distinct, supporting the results of the ImageJ/Fiji analysis.

### PROX1

As PROX1 is a specific marker for lymphatic endothelial cells, it serves to identify lymphatic vessels in tissue samples. The presence of numerous lymphatic endothelial cells indicates active and essential processes of tissue repair, regeneration and immune surveillance. PROX1-specific cells were detected to varying degrees in all experimental groups. Similar to the factor VIII and SMA staining, lymphatic endothelial cells were randomly distributed and attached to the BTM structure when stained with PROX1. This suggests that the PROX1 antibody effectively highlights lymphatic endothelial cell activity, although the ratio of lymphatic to non-lymphatic endothelial cells within each experimental group was not quantified.

## Discussion

To the authors’ knowledge, the study at hand represents one of the first description of an optimized vascularization of a dermal skin substitute in ovo by adding recombinant growth factors and/or ASC. Skin substitutes have been available on the market for many years and have become an integral part of reconstructive surgery, especially in the treatment of severe burns [29, 30]. BTM is one of the most commonly used dermal skin substitutes and has been shown to improve functional and aesthetic outcomes in many studies [31]. As BTM is a fully synthetic dermis substitute material, ethical and cultural issues that arise when using biological or biosynthetic dermis substitute materials can be avoided [32]. As described elsewhere, other adverse events such as infection, allergy, or excessive scarring tend not to occur [33, 34]. For example, BTM was used in 10 patients to reconstruct the donor sites of free flap plasty, with only minor scarring observed [35, 36]. Further studies successfully applied BTM for the reconstruction of complex wounds in patients with multiple co-morbidities [37, 38]. Despite these convincing clinical results, however, the prolonged time for successful integration and vascularization, which can take up to 21 days, has proven to be problematic. The associated costs for prolonged hospitalization, subsequent rehabilitation, and long-term absence from work generate a significant socioeconomic burden [39–41]. It was assumed that 1% burned TBSA costs an average of $4159 in high-income countries [42]. In addition to the socioeconomic burden, burns have a significant impact on patients’ quality of life, potentially leading to immobility, psychosocial stress and chronic pain [43]. The evidence supporting the use of BTM is compelling, with numerous studies confirming the proangiogenic effect of ASC and the growth factors employed [44, 45]. In the present study, BTM was treated with ASC or with individual growth factors (EPO, FGF, PDGF, VEGF) or a combination of ASC and a growth factor. ASC have the potential to form bone, cartilage, muscle, and fat tissue, representing an exciting perspective for regenerative medicine and surgery [46, 47]. It has been previously described that ASC contribute to fat tissue turnover, enhance vascularization and secrete cytokines that accelerate neoangiogenesis and wound healing [48, 49]. ASC are also an autologous patient material, providing a simple and immunologically safe source for reconstructive procedures [28]. Several studies have focused on the application of ASC in combination with the aforementioned recombinant growth factors, as they promote proliferation, growth, and migration of epithelial cells [50–57]. The proliferation and migration ability of ASC correlated in most cases with the given amount of growth factors [58].

In the present study, grafting and vascularization of BTM were observed in all experimental groups after a period of 7 days. Various methods, ImageJ/Fiji as well as HE and immunohistochemical staining, were used to analyze successful vascularization in the experimental groups compared to the control group. Despite this, none of the experimental groups demonstrated statistically significant increases in vessel density compared to control. The lack of statistical significance limits the strength of these findings and may be attributable to the relatively small sample sizes per group and the wide inter-group variability inherent in the CAM model. This issue is compounded by the large number of treatment combinations, which, while conceptually informative, dilute statistical power and increase the risk of type I error. The underlying principle of this study is predicated on the notion that it functions as a proof-of-concept model. In this capacity, it is posited that initially the most substantial augmentation in knowledge that can be attained for future experiments is to be realized with the most minimized number of experiments. Another important consideration is the anatomical focus of vascular analysis. Although the primary objective was to evaluate the vascular integration of the BTM itself, much of the vascular signal—both macroscopically and histologically—was derived from the surrounding CAM tissue. This raises the possibility that BTM exerts indirect proangiogenic effects on adjacent tissue or that the observed responses reflect a broader systemic reaction within the egg. However, in the absence of a CAM-only control group without BTM, we are unable to definitively disentangle local matrix-associated angiogenesis from more diffuse effects.

Although the following observations should be interpreted with caution due to a lack of statistical significance, different trends were observed in the ImageJ/FIji analysis, including overall CAM vascularization, with the control group showing a lower vessel density than all other experimental groups. This could be the first indication that ASC and the selected growth factors EPO, FGF, PDGF and VEGF exert a pro-angiogenic effect. In the ImageJ/Fiji analysis of the entire CAM, the BTM + ASC experimental group showed the most pronounced vascularization. However, in the ImageJ/Fiji analysis, which only referred to BTM, this statement no longer applied. It was also shown that ASC in combination with individual growth factors appeared to retain its pro-angiogenic effect compared to the control group. The fact that PDGF produces particularly striking results cannot yet be clearly explained and requires further investigation. Histological and immunohistochemical findings further supported the presence of endothelial cells and vascular-like structures across all groups. However, these findings remain descriptive, and no quantitative analysis of staining intensity, vessel cross-section density, or positive cell counts was performed. This represents a limitation, as subjective interpretation may lead to over- or underestimation of biological relevance.

It is important to note that the present study investigated the lowest number of growth factor concentrations and a predetermined number of 25,000 ASC per egg. This number was chosen to balance biological efficacy, ensuring an adequate number of viable cells for local interaction, taking the physical limitations of the CAM environment into account. However, a dose-response study was not performed in the current study, and the optimal ASC number for inducing angiogenesis remains undefined. Previous experiments have shown that CAM vascularization does not increase proportionally to the amount of growth factors, but rather peaks [59]. Moreover, projects in which growth factors were administered in combination should also be tested on BTM in the CAM model.

The CAM model, which has become well-established in regenerative medicine, is suitable for studying the vascularization of various biomaterials and growth factors [60]. CAM allows for the observation of pro- and anti-angiogenic molecules, tissue grafts, tumor growth, drug delivery, metastasis, and toxicological analyses [60]. The model is simple, inexpensive, and quick to implement [60, 61]. The stages of CAM development have been described according to Hamilton and Hamburger, so the timing of application of external agents and their effects are standardized [62]. The chick embryo’s immune system becomes active around 4 days before hatching, allowing it to receive grafts from different tissues and species without graft rejection [63–65]. Moreover, the influence of various growth factors on neovascularization in CAM has been investigated [59, 66, 67].

There is also an autologous option in the form of platelet-rich plasma (PRP), which contains a combination of the growth factors FGF, PDGF and VEGF used in this study. PRP is easy and inexpensive to obtain [68, 69]. PRP has been successfully applied in burn patients in various trials, making it a promising therapeutic option in combination with BTM as a lead structure. [43, 70]. It is known that specific cytokines and growth factors are required at each stage of wound healing [71]. Consequently, the optimal timing of topical application should be discussed.

In the present study, administration was always performed at EDD 10 for better comparability. Further investigations are required to determine the influence of timing on vascularization [72]. Lymphogenic infiltration was also detected in the immunohistochemical staining for PROX1. As an immunosuppressed carrier, CAM does not allow a clear assessment of the extent to which a lymphogenic reaction to BTM, growth factors, or stem cells would occur in humans. If autologous supplements are used such as PRP, this aspect could be disregarded. Notably, in the PROX1 results, that the BTM + FGF and BTM + FGF + ASC groups, which were weakly vascularized in the ImageJ/Fiji and HE analyses, showed fewer lymphatic endothelial cells. The opposite was true for the BTM + PDGF and BTM + PDGF + ASC groups, which tended to exhibit higher levels of vascularization. Since the lymphatic activity, associated with defense and inflammatory reactions, promotes to neoangiogenesis, further research at this point seems promising [73, 74].

Overall, while the current study does not yield statistically significant evidence of improved vascularization through any specific proangiogenic adjunct, it offers important insights into the biological behavior of BTM within a vascularized environment. The observed trends, particularly with PDGF and ASC, may warrant further investigation under more controlled and statistically powered conditions. Moreover, the feasibility of BTM integration and the presence of molecular hallmarks of angiogenesis across multiple groups support its candidacy as a vascularizable dermal matrix for clinical applications, especially in the context of complex wounds or extensive burns. Moving forward, we suggest that research efforts refocus on a more granular analysis of BTM integration as a baseline, rather than attempting to screen multiple combinatorial enhancement strategies simultaneously. Such an approach would enable clearer mechanistic insights into BTM-host tissue interaction and better inform clinical translation strategies for its optimized use in regenerative surgery. Based on existing clinical approaches, such as with PRP, a timely transfer to clinical application would be feasible. Nevertheless, further in ovo and in vivo trials should be carried out to support the current data.

## Conclusions

In summary, the presented results suggest that the application of growth factors and ASC can be a beneficial addition to BTM therapy in clinic settings. Specifically, the combination of ASC + PDGF appears promising, taking into account the statistical limitations. When comparing with a wide range of dermal synthetic skin substitutes, investigating integration and vascularization in the CAM model proves highly suitable for investigating questions regarding growth factor concentrations and combinations, as well as PRP application, among other factors.

## Supplementary information

Supplementary Materials

## Data Availability

The datasets used and/or analyzed during the current study are available from the corresponding author upon reasonable request.

## Abbreviations

ASC: adipose derived stromal cells
BSA: bovine serum albumin
BTM: Biodegradable Temporizing Matrix
CAM: chorioallantoic membrane
DAPI: 4′,6-diamidino-2-phenylindole
DMEM/F-12: Dulbecco´s Modified Eagle Medium
EDD: embryonic development day
EGF: endothelial growth factor
EPO: erythropoietin
FDA: US Food and Drug Administration
FGF: fibroblast growth factor
HE: hematoxylin/eosin
PBS: phosphate buffered saline
PDAF: platelet-derived angiogenesis factor
PDGF: platelet-derived growth factor
PROX1: prospero homebox protein 1
PRP: platelet rich plasma
SMA: alpha smooth muscle actin
TBSA: total body surface area
VEGF: vascular endothelial growth factor
