# Supplementary material for: Vascularization potential of a dermal skin substitute material (Biodegradable Temporizing Matrix) by proangiogenic growth factors and ASC - an in ovo study
Source: J Mater Sci Mater Med. 2025 Dec 13;37(1):6. doi: 10.1007/s10856-025-06982-4 (PMC12714854; doi:10.1007/s10856-025-06982-4)
Supplement: Supplementary file 1 — Supplementary Materials [file 10856_2025_6982_MOESM1_ESM.docx]

**Supplementary Materials
Supplementary Figures:**

**Supplementary Figure 1:** Analysis of BTM vascularization on the CAM. A photo of the BTM *in ovo* was recorded and analyzed using ImageJ/Fiji vascularization filters. The vascularization of both the BTM and the entire CAM, including the BTM, was calculated. In this case, a BTM vascularization of 49.6% and a total vascularization of 70.0% was observed.


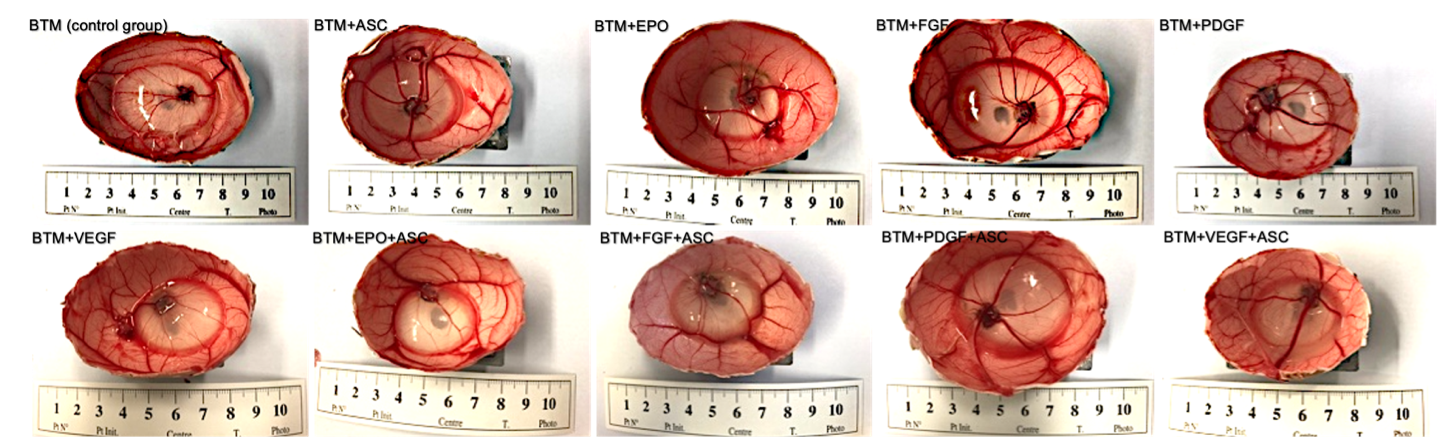


**Supplementary Figure 2:** One example of each experimental group showing that the BTM that was successfully transplanted onto the CAM and displayed varying degrees of vascularization.
